# Socioeconomic inequality in public satisfaction with the healthcare system in China: a quantile regression analysis

**DOI:** 10.1186/s13690-022-00925-z

**Published:** 2022-07-08

**Authors:** XinXin Peng, Jing Hua Zhang

**Affiliations:** 1 School of Business, Macao University of Science and Technology, Macao S.A.R., China; 2 School of Business, Jiangsu University of Technology, Changzhou, China

**Keywords:** Socioeconomic inequality, Public satisfaction, Healthcare system, Quantile regression

## Abstract

**Background:**

As China pursues better social equality and improvement in public services (healthcare), public satisfaction has been considered as a key performance indicator. There is a great need to better understand the disparities and inequalities in the public satisfaction with its healthcare system.

**Methods:**

Based on Chinese General Social Survey (CGSS) 2015 ( a set of nationally representative survey data, the most recent wave containing information about public satisfaction with the healthcare system), this study utilizes the quantile regression method to analyze how the public satisfaction at high or low quantile of the score distribution varies according to the socio-economic status and healthcare system performance indicators, especially in rural areas.

**Results:**

This study found that, at the highest percentile, better Self-Reported-Health (SRH) is associated significantly with a lower satisfaction score (coefficient -4.10, *P* < 0.01). High socioeconomic status (especially "above average" group) has higher satisfaction scores at both mean (coefficient 3.74, *P*<0.01) and median (coefficient 3.83, *P<*0.01). This effect is also significant across the lower quantiles of the satisfaction levels. West and Middle region (the less developed regions) tended to be more satisfied, whereas those in Northeast reported a large negative effect (coefficient -7.07, *P* < 0.01) at the median. While rural residents generally reported higher levels of satisfaction, rural residents’ preference regarding hospital beds and primary care access seems generally to be opposite to that of urban residents.

**Conclusion:**

Our findings suggest that the ongoing healthcare reform needs to integrate more preventive care to meet the healthy residents’ expectation and demands. More attention should be guided to the vulnerable healthcare system in the Northeast region, which has a stagnant local economy. Outcome-based quality care is especially preferred in rural healthcare, in addition to improvement of utilization and access. In addition, the “pro-rich” inequality is an ongoing concern about the system.

## Introduction

### Public satisfaction with the healthcare system

Public satisfaction with the healthcare system measures the satisfaction of general residents who are qualified for the system, regardless of the direct utilization of care provided by the system [1–3]. The major determinants of satisfaction level include the key performance indicators of a healthcare system, such as accessibility, quality of care, and affordability [1, 4–7]. Meanwhile, it is also influenced by residents’ personal expectations, demographic and socioeconomic characteristics, as well as the social media environment [2, 3, 8–10]. Owing to these features, public satisfaction has been regarded as an important indicator for monitoring the performance of a nation’s healthcare system [3, 6, 11], as well as a key measure of government administration performance [4] and social welfare [12]. Meanwhile, significant variations among the association factors have been observed across countries [13, 14]. Populations with financial and health vulnerabilities were found to have lower level of satisfaction with the healthcare system even among European countries [13].

### China’s health system, resource disparities, and public satisfaction

The healthcare system in China is a public hospital-based delivery system [15]. The hospital accreditation system has three levels, with tertiary hospitals having the best service quality and most advanced medical technology [5, 7, 16–19]. Due to long-time geographical disparities and social economic policy reasons, all tertiary hospitals concentrate in central cities [20–22]. The healthcare workforce densities and reimbursement rates of social health insurance among urban residents are about twice those of rural residents [23]. Among the fast economic growth and dramatic social changes during the past decades, there have been rising demands and expectations for better healthcare services, while inequalities in health care have further expanded [24]. The unmet demands or dissatisfactions of the public are well reflected in a widely cited public expression that “seeing a doctor is hard, seeing a doctor is expensive” [25]. Some patients and their family tried to obtain access of high-quality healthcare through tapping on their social networks or even bribery [24]. Peaking around 2015, the dissatisfaction about the healthcare system in China was also reflected in the intense physician–patient relationships and even physical violence towards healthcare staff [20, 24]. Since then, the importance of public satisfaction has received increased attention in China [1, 9, 11] and been included in the main goals of the ongoing health policy reform. The Common Prosperity, a new national strategic campaign launched by the Chinese government in August 2021, has set public satisfaction and equality of healthcare as key strategic objectives too.

Despite the rising volume of literature studying the satisfaction of the general population in China [26–29], none focuses on the socioeconomic inequality of the satisfaction or the disparities among social vulnerability factors in China. By estimating the average effects, current conventional regression methods ignore the potential heterogeneity or disparities across quantiles of the distribution of dependent variables. There is no comprehensive examination or understanding about the distribution of inequality in the public satisfaction in China [30, 31].

### Aims of this study

Applying quantile regression method, this study intends to examine the potential inequality of public satisfaction with the healthcare system in China. The quantile regression method can provide detailed information about the differentiated preferences and needs of residents across various levels of satisfaction. Specifically, this study aims to analyze: (1) how the public satisfaction at high or low quantiles of the distribution varies according to socio-economic characteristics and healthcare system performance indicators, and (2) how public satisfaction at various quantiles differs with the relevant factors among rural residents in China. With this detailed information, policymakers will be able to respond with precision or intervene in more effective and efficient ways [32, 33].

## Methods

### Data source and sample

Chinese General Social Survey (CGSS) is a nationally representative survey conducted in mainland China. The CGSS 2015 was adopted for this study because it is the most recent wave of the CGSS that contains a survey question about public satisfaction with the healthcare system in China. The CGSS adopted a multi-stage stratified sampling design, with counties as the primary sampling unit (PSU). Totally 2762 PSUs nationwide were included in the sampling frame, and 12,000 households were sampled. The sampling weights were based on the total population parameters of the survey year. Following the KISH grid process, adults (18 years or older) from each household were randomly selected for face-to-face family interviews. After excluding those with missing values or incomplete answers, the final sample contained 10,433 respondents.

The healthcare system measurement indicators were obtained from the China Public Health Statistical Yearbook 2015. The data were aggregated at the provincial level.

Ethics approval was not applicable for this study because anonymized secondary data were used.

### Measurement and variables

The dependent variable was the public satisfaction score with the healthcare system in China, while the explanatory variables included the respondents’ demographic and socioeconomic characteristics, self-reported health status, and a set of healthcare system performance indicators aggregated at the provincial level.

The satisfaction score was collected through a survey question, asking “Taking all aspects into consideration, what is your general satisfaction with the healthcare system?” Respondents need to be evaluated based on their personal experience and assigned a score ranging from 0 to 100 points, where “100” means perfectly satisfied.

The demographic characteristics were as follows: senior group (≥ 60 years), gender (male = 1), marital status (cohabitation/married = 1), and ethnic group (Han = 1). Socioeconomic information included education level (a category variable), living in a rural area (yes = 1), internal migrant status (yes = 1), employment status (employed/working on farms = 1), primary health insurance status (with any health insurance = 1). Self-reported socioeconomic status is based on the answer to a survey question, which asked "In your living community, which socioeconomic status do you think you and your family belong to?" (“far below average” = 1, “below average” = 2, “average” = 3, “above/far above average” = 4).

Self-Reported Health (SRH) status was measured on a 5-point Likert scale (very unhealthy = 1, unhealthy = 2, so-so = 3, healthy = 4, and very healthy = 5). In addition, the Subjective-Wellbeing (SWB) took the value of “1,” if being generally satisfied with life [8].

As for the healthcare system performance indicators, bed occupancy and daily visits per physician in public hospitals were adopted to measure healthcare accessibility of inpatient and outpatient service respectively[34]. Healthcare expenditure/disposable income per capita was adopted to measure the affordability or the economic burden of healthcare. Aggregated on the provincial level, the values in urban and rural areas were included respectively. A set of regional dummy variables was included to control for differences due to regional economic development [9, 30]. Eight standard economic regions or municipalities in mainland China were included (East, Middle, West, Northeast, Beijing, Shanghai, Tianjin, and Chongqing).

### Statistical method

The quantile regression method generates estimates of a conditional quantile function by minimizing the sum of asymmetrically weighted absolute residuals [35, 36]. This method provides several advantages. First, making no assumption about the distribution of the underlying data, quantile regression provides unbiased estimates, even in the presence of non-normal distribution or outlier observations [27, 31, 36, 37], which are often common among healthcare cost [38] or utilization data [39]. Indeed, the public satisfaction scores with health care usually do not have a normal distribution either [30, 31]. Additionally, the quantile regression approach provides snapshots of different points in a conditional distribution; therefore, a comprehensive picture of the relationships can be obtained [36].

The baseline model in this study is a multivariate equation that analyzes the association between satisfaction scores of the healthcare system and major relevant factors at various percentiles of the dependent variable. To identify potential socioeconomic disparities, interaction terms of rural areas with relevant indicators were constructed and estimated.

The command *sqreg* in the Stata 15 statistical package (Stata Corp LP, College Station, TX, USA) estimates simultaneous quantile regression and produces the same coefficients as *qreg* for each quantile. Standard errors were calculated by bootstrapping with 200 repetitions. The regressions were performed at the 10^th^, 30^th^, 50^th^, 80^th^, and 90^th^ percentiles because of the unsmooth distribution of the data. OLS regressions were also performed for benchmarks.

## Results

**Fig. 1:**
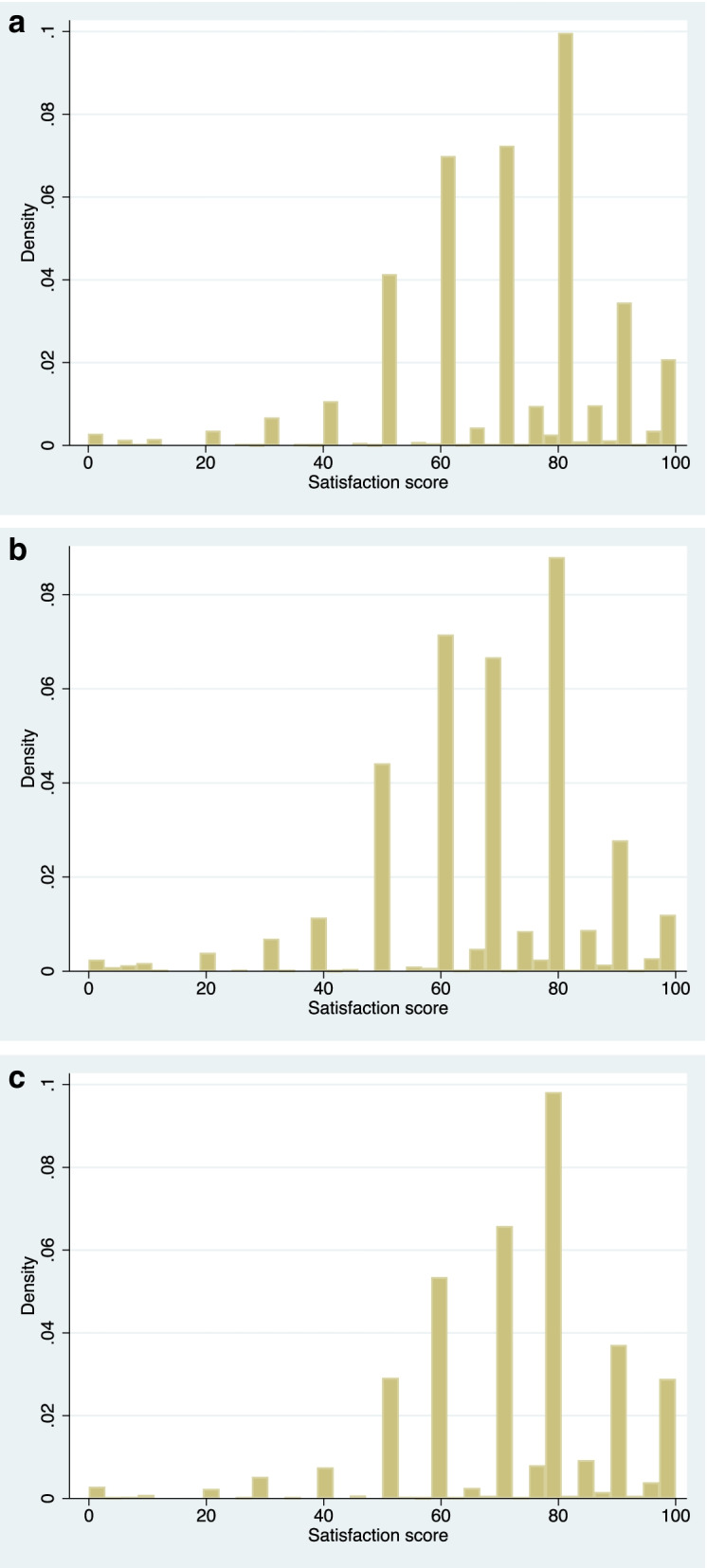
The distribution of the satisfaction score of the healthcare system in China, CGSS, 2015, China (*n* = 10,433)

As shown in Fig. 1, the satisfaction scores in this study did not have a normal distribution. In addition, the data values of the score tended to cluster at points that were multiples of 10. As a result, the distribution of the scores was not continuous.

As shown in Table 1, the mean satisfaction score in the full sample was 69.75, while that in rural areas was 72.53. In total, 4,303 respondents (41.24%) were rural residents. About 64% of urban residents reported being “healthy” or “very healthy,” whereas only about 55.7% of rural residents reported so. In total, about 8.4% of all respondents (10.21% urban and 5.81% rural) reported socioeconomic status as “above average”.

**Table 1:** Descriptive statistics of the demographic and socioeconomic characteristics, CGSS, 2015, China

	**Full sample**	**Urban**	**Rural**	**χ**^**2**^	***p*-value**	**Satisfaction score**	
	**%**	**%**	**%**			**Mean**	**SE**
**Observation numbers**	10,433	6,130	4,303	-	-	-	-
**Satisfaction score**	69.75	67.80	72.53	-	-	69.75	0.18
**Age**							
Age (average years)	50.21	48.87	52.12	-	-	-	-
Age < 60 (%)	67.65	70.10	64.16	40.67	< 0.001	68.90	0.22
Age > = 60 (%)	32.35	29.90	35.84			71.52	0.30
**Gender (%)**							
Male	52.99	53.59	52.13	2.17	0.141	70.08	0.24
Female	47.01	46.41	47.87			69.38	0.26
**Ethnic group (%)**							
Han	92.25	94.57	88.94	112.03	< 0.001	71.92	0.63
Ethnic minority	7.75	5.43	11.06			69.57	0.18
**Marital status (%)**							
Single/separated/widow/widower	21.61	23.77	18.55	40.71	< 0.001	69.21	0.38
Cohabitation/married	78.39	76.23	81.45			69.90	0.20
**Employment status (%)**							
Not working	42.44	47.05	35.88	129.02	< 0.001	70.30	0.27
Employed/Farm	57.56	52.95	64.12			69.34	0.23
**Education (%)**							
Elementary School or less	37.07	23.13	56.91	1600.00	< 0.001	71.93	0.29
Middle/high School	46.58	51.62	39.41			69.00	0.26
College	15.35	23.61	3.58			67.35	0.41
Postgraduate	1.01	1.65	0.09			60.70	1.74
**Self-reported health (%)**							
Very unhealthy	3.04	2.06	4.44	212.30	< 0.001	67.79	1.28
Unhealthy	14.63	11.09	19.66			69.62	0.49
So-so	21.59	22.58	20.20			69.40	0.38
Healthy	39.07	41.04	36.25			69.89	0.27
Very healthy	21.67	23.23	19.45			70.20	0.38
**Self-reported household socioeconomic status (%)**							
Far below average	5.36	4.19	7.02	109.03	< 0.001	66.83	0.90
Below average	32.12	30.59	34.30			68.03	0.32
Average	54.13	55.01	52.87			70.87	0.22
Above average	8.40	10.21	5.81			70.97	0.59
**Insurance status (%)**							
Without any health Insurance	9.00	10.36	7.06	33.50	< 0.001	66.83	0.62
With any health insurance	91.00	89.64	92.94			70.04	0.18
**Internal migrant (%)**							
No	89.97	84.55	97.70	484.50	< 0.001	70.28	0.18
Yes	10.03	15.45	2.30			65.00	0.58
**Subjective-wellbeing (%)**							
Not good	21.90	21.17	22.94	4.59	0.032	66.15	0.40
Good	78.10	78.83	77.06			70.76	0.19
**Regions or municipalities**							
East China (without Shanghai)	23.86	27.39	18.82	1300.00	< 0.001	68.75	0.35
Middle China	26.15	22.09	31.93			72.18	0.31
West China (without Chongqing)	22.40	16.25	31.16			72.23	0.35
NEast China	13.26	13.18	13.36			64.75	0.56
Beijing	4.94	7.98	0.60			66.96	0.86
Shanghai	4.30	7.32	0.00			64.71	0.87
Tianjin	2.68	4.57	0.00			67.50	1.08
Chongqing	2.42	1.22	4.11			74.90	0.88

Table 2 reports the descriptive statistics of some key performance indicators of the healthcare system in China (2015). In rural areas, the daily visits per physician in public hospitals (6.72) are lower than those in urban areas. Meanwhile, the healthcare burden is larger in rural areas.

**Table 2:** Descriptive statistics of Chinese healthcare system performance indicators (*China Public Health Statistical Yearbook*, 2015, China)

	**Full sample**				**Urban**				**Rural**			
	Mean	95% CI			Mean	95% CI			Mean	95% CI		
**Public hospital beds occupancy (%)**	90.54	90.45	to	90.62	90.79	90.68	to	90.91	90.17	90.04	to	90.30
**Daily visits per physician in public hospitals**	7.77	7.71	to	7.82	8.50	8.42	to	8.58	6.72	6.67	to	6.77
**Healthcare expenditure/disposable income per capita (%)**	6.58	6.54	to	6.62	5.68	5.64	to	5.73	7.86	7.81	to	7.91

The healthcare system performance indicators are on provincial level. Totally 31 provinces or municipalities in mainland China were included

### Disparities in the associated factors across quantiles in the baseline model

Table 3 reports the results of quantile regression analysis using the baseline model. Column (1) reports the coefficients estimated by OLS, and Columns (2) to (6) report those of the quantile regression. In Table 3, the coefficients of most demographics and socioeconomic variables (except the SRH status groups) have consistent signs when estimated at lower or higher percentiles, despite variations in size. Rural residents consistently reported higher satisfaction scores when estimated by OLS (coefficients 2.65, *P* < 0.01) or quantile regressions (coefficients from 2.2 to 3.6, *P* < 0.01). While no significant results were estimated with OLS, when estimated at the 10^th^ percentile (among the least satisfied residents), the satisfaction scores of better SRH groups were significantly higher by about 7.98 points (“Unhealthy”) to 10.92 points (“very healthy”), compared with the reference group of being “Very unhealthy”. However, at the 90^th^ percentile (among the residents with high level satisfaction), the scores of the better SRH groups were significantly lower by approximately 4 to 5.85 points.

**Table 3:** Quantile regression analysis of public satisfaction with the healthcare system (baseline model), CGSS, 2015, China. Dependent variable: Satisfaction score

VARIABLES	(1)	(2)	(3)	(4)	(5)	(6)
	OLS	Q10	Q30	Q50	Q80	Q90
**Senior60**						
Age < 60	Reference					
Age > = 60	1.42***	1.89**	1.89***	0.95*	1.01	0.67
	(0.45)	(0.89)	(0.52)	(0.51)	(0.63)	(0.64)
**Gender**						
Male	Reference					
Female	-0.35	-0.55	-0.04	-0.05	-0.25	-0.96**
	(0.35)	(0.72)	(0.44)	(0.35)	(0.34)	(0.47)
**Ethnic group**						
Han	Reference					
Ethnic minority	-1.30*	-1.94*	-1.07	-1.10	-1.25	-2.68***
	(0.69)	(1.01)	(0.83)	(0.75)	(1.03)	(0.93)
**Marital status**						
Single/separated/widow/widower	Reference					
Cohabitation/married	-0.24	0.03	-0.44	-0.30	-0.19	0.17
	(0.43)	(0.87)	(0.55)	(0.38)	(0.35)	(0.55)
**Employment status**						
Not working	Reference					
Employed/Farm	-0.93**	-0.02	-0.68	-1.21***	-0.83*	-0.93*
	(0.40)	(0.79)	(0.49)	(0.43)	(0.50)	(0.56)
**Education**						
Elementary School or less	Reference					
Middle / high School	-1.33***	-1.78*	-1.54***	-1.37***	-0.93	-1.66***
	(0.44)	(0.93)	(0.54)	(0.50)	(0.57)	(0.62)
College	-2.12***	-0.31	-1.75**	-3.15***	-1.77**	-3.53***
	(0.62)	(1.34)	(0.80)	(0.76)	(0.71)	(0.91)
Postgraduate	-7.73***	-13.07**	-8.15***	-6.01**	-4.14*	-8.80***
	(1.78)	(5.19)	(2.26)	(2.87)	(2.33)	(1.80)
**Self-reported health**						
Very unhealthy	Reference					
Unhealthy	0.79	7.98**	1.15	1.30	-2.63	-4.42***
	(1.34)	(3.69)	(1.71)	(1.54)	(2.42)	(1.23)
So-so	1.64	10.59***	2.98*	1.87	-3.19	-5.40***
	(1.32)	(3.56)	(1.63)	(1.58)	(2.22)	(1.13)
Healthy	1.96	10.54***	3.85**	2.32	-2.99	-5.85***
	(1.31)	(3.43)	(1.66)	(1.56)	(2.31)	(1.14)
Very healthy	2.68**	10.92***	4.11**	3.77**	-1.97	-4.10***
	(1.35)	(3.51)	(1.66)	(1.63)	(2.35)	(1.19)
**Self-reported household economic status**						
Far below average	Reference					
Below average	0.55	1.62	0.54	0.35	-0.68	-1.02
	(0.96)	(2.43)	(1.22)	(1.15)	(0.93)	(1.21)
Average	2.89***	5.06**	2.90**	2.73**	0.53	0.86
	(0.96)	(2.43)	(1.22)	(1.20)	(0.93)	(1.25)
Above average	3.74***	4.74*	4.11***	3.83***	1.62	2.32
	(1.10)	(2.64)	(1.40)	(1.19)	(1.24)	(1.45)
**Insurance status**						
Without any health Insurance	Reference					
With any health insurance	1.24*	2.34	0.74	1.42**	0.62	0.80
	(0.64)	(1.57)	(0.75)	(0.69)	(0.61)	(1.12)
**Internal migrant**						
No	Reference					
Yes	-2.40***	-1.86	-2.83***	-3.60***	-0.83*	-1.46
	(0.63)	(1.32)	(0.69)	(0.82)	(0.49)	(0.91)
**Subjective-wellbeing**						
Not good	Reference					
Good	3.93***	5.29***	4.47***	5.36***	1.85***	2.65***
	(0.46)	(1.08)	(0.63)	(0.61)	(0.60)	(0.66)
**Rural residency**						
Urban	Reference					
Rural	2.65***	2.68***	2.66***	2.17***	3.55***	2.77***
	(0.44)	(0.90)	(0.70)	(0.61)	(0.94)	(0.59)
Public hospital beds occupancy	0.18***	0.38***	0.22***	0.17***	-0.04	-0.19***
	(0.05)	(0.09)	(0.07)	(0.05)	(0.04)	(0.07)
Daily visits per physician in public hospitals	-0.50***	-0.22	-0.35*	-0.47***	-0.36*	-0.41***
	(0.13)	(0.25)	(0.18)	(0.16)	(0.21)	(0.15)
Healthcare expenditure/ disposable income	0.19	-0.23	0.22	0.38***	0.18	0.21
	(0.13)	(0.28)	(0.16)	(0.13)	(0.14)	(0.17)
**Regions or municipalities**						
East China (without Shanghai)	Reference					
Middle China	0.79	4.81***	3.27***	1.99**	-1.31	-1.80**
	(0.61)	(1.11)	(1.11)	(0.80)	(1.07)	(0.76)
West China (without Chongqing)	1.19*	4.42***	2.95***	1.69**	0.25	-0.13
	(0.63)	(1.05)	(1.02)	(0.85)	(1.05)	(0.71)
NEast China	-5.82***	-5.45***	-5.83***	-7.07***	-3.22**	-2.35*
	(0.83)	(1.79)	(1.21)	(0.98)	(1.41)	(1.25)
Beijing	1.84*	0.71	0.33	1.13	1.88	2.80**
	(0.99)	(1.69)	(0.92)	(1.34)	(1.35)	(1.17)
Shanghai	-0.50	-1.60	0.09	0.07	1.13	-0.81
	(1.23)	(2.64)	(1.58)	(1.21)	(1.06)	(1.35)
Tianjin	1.00	1.08	2.45	1.04	-0.51	0.64
	(1.21)	(4.28)	(1.79)	(1.25)	(0.61)	(2.14)
Chongqing	4.38***	10.04***	5.64***	4.13***	0.42	-0.37
	(0.97)	(1.35)	(1.37)	(0.88)	(1.45)	(1.76)
Constant	50.19***	-2.10	35.59***	49.79***	89.82***	115.26***
	(4.50)	(8.79)	(6.03)	(4.71)	(4.24)	(6.00)
Observations	10,433	10,433	10,433	10,433	10,433	10,433
R-squared / pseudo R-squared	0.07	0.028	0.032	0.035	0.015	0.021

(1) *** *P* < 0.01, ** *P* < 0.05, * *P* < 0.1

(2) Robust standard errors in parentheses

(3) R-squared is not applicable for quantile regressions and pseudo R-squared was calculated as reference information

When estimated at mean and median, higher socioeconomic status (both “Average” and “above average” group) is associated with significantly higher satisfaction scores when compared with those with social status of “Below average” or “Far below average.” The effects are especially significant among the satisfaction levels at median or lower quantiles.

As reported in the lower section of Table 3, the quantile regression estimates of the healthcare system performance variables indicated large disparities and changes when moving across the conditional distribution of the public satisfaction score. While being positive when estimated by OLS, the coefficients of “public hospital bed occupancy” have changed from being positive at the lower percentiles to being negative at the 90^th^ percentile. The coefficients of “Daily visits per physician in public hospitals” are mostly negative by both OLS and quantile regression. The ratio of healthcare expenditure/disposable income is only significant at the median with a positive coefficient of 0.38 points (*P* < 0.1).

Compared with those in the eastern region (the coastal region, the most developed region) of China, those at median and lower quantiles in the Middle, West, and Chongqing (the less developed regions) tended to report significantly higher satisfaction scores, ranging from about 2 to 4.8 points (*P* < 0.01). In Northeast China, the satisfaction score was estimated to be lower by 5.8 points on average or about 7 points at median respectively.

### Disparities in the key performance indicators among rural residents

Table 4 reports the results with a focus on those in rural areas. The first line of Table 4 indicates that, with the full set of covariates controlled, higher levels of satisfaction in the rural area are large and consistent, except the 10^th^ percentile (the least satisfied).

**Table 4:** Quantile regression analysis of public satisfaction with the healthcare system in rural areas, CGSS, 2015, China. Dependent variable: Satisfaction score

VARIABLES	(1)	(2)	(3)	(4)	(5)	(6)
	OLS	Q10	Q30	Q50	Q80	Q90
**Rural residency**						
Urban	Reference					
Rural	34.67***	17.51	32.24***	33.53***	52.69***	49.72***
	(8.30)	(20.31)	(11.70)	(10.40)	(9.44)	(11.77)
Public hospital beds occupancy	0.31***	0.46***	0.36***	0.34***	0.08	0.08
	(0.06)	(0.16)	(0.10)	(0.09)	(0.05)	(0.09)
Daily visits per physician in public hospitals	-0.66***	-0.33	-0.44*	-0.70***	-0.38	-0.55***
	(0.14)	(0.31)	(0.23)	(0.21)	(0.24)	(0.20)
Healthcare expenditure/ disposable income	0.24	0.14	0.27	0.37	0.09	-0.09
	(0.16)	(0.39)	(0.25)	(0.23)	(0.14)	(0.18)
Public hospital beds occupancy*Rural residency	-0.41***	-0.15	-0.39***	-0.41***	-0.63***	-0.57***
	(0.09)	(0.22)	(0.13)	(0.11)	(0.12)	(0.13)
Daily visits per physician in public hospitals*Rural residency	0.81***	0.43	0.96**	1.04***	0.90**	0.44
	(0.24)	(0.48)	(0.38)	(0.31)	(0.39)	(0.30)
Healthcare expenditure/ disposable income*Rural residency	-0.12	-0.57	-0.08	-0.12	0.25	0.36
	(0.24)	(0.55)	(0.32)	(0.28)	(0.36)	(0.32)
Constant	38.81***	-10.81	22.72***	36.14***	81.37***	93.15***
	(5.43)	(14.26)	(8.06)	(7.60)	(4.61)	(7.01)
Observations	10,433	10,433	10,433	10,433	10,433	10,433
R-squared / pseudo R-squared	0.07	0.029	0.033	0.037	0.018	0.025

(1) *** *P* < 0.01, ** *P* < 0.05, * *P* < 0.1

(2) Robust standard errors in parentheses

(3) R-squared is not applicable for quantile regressions and pseudo R-squared was calculated as reference information

(4) Not reported here, the regressions in Table 4 have controlled all other variables (including demographics, socioeconomics, self-reported health, subjective well-being, region dummies) as listed in Table 3

As shown in Table 4, for public hospital beds occupancy and daily visits per physician in public hospitals, the estimated coefficients among the urban and rural respondents demonstrated opposite direction of changes in satisfaction scores. While urban respondents seemed to prefer more access to public hospital beds (at mean and median) and less daily visits per physician (at mean, median and highest quantile) in public hospitals, rural respondents demonstrated opposite direction for their satisfaction.

When examined separately among urban and rural respondents, the effects of economic burden of healthcare are not significant in Table 4.

## Discussion

This study utilizes the quantile regression method to analyze the public satisfaction score of China’s healthcare system based on a set of nationally representative survey data (CGSS 2015). Unlike the simple positive association between SRH and the satisfaction with health system [2, 3, 17, 18, 40] when estimated at mean, the quantile analysis results in this study found that better SRH status had a large and significant negative association at the 90^th^ percentile (the highest) satisfaction scores. Because they are generally very healthy, the healthcare demand of these groups may mainly be preventive [34]. Still largely focusing on disease treatment, the current healthcare system in China has not yet provided satisfactory preventive care and coverage for the healthy group. Meanwhile, among the least satisfied, those with good SRH reported higher satisfaction scores, possibly because of less unmet demand for care. These heterogeneous associations between SRH and satisfaction with the healthcare system have not yet been reported or identified in the existing literature.

Concerns about “pro-rich” inequality in the Chinese healthcare system have been well documented [27]. With more economic and social capital resources, higher socioeconomic status in China could have good access to and utilization of high-quality public healthcare resources [20, 27]. The “pro-rich” inequality was especially significant among those at median and lower quantile of satisfaction levels.

Analysis of this study on regional disparities indicates that the higher satisfaction scores in the West and Middle region (the less developed regions) were mainly among the lower percentiles, who might be the healthcare disadvantaged groups and have benefited from the healthcare reform programs of enhancing primary care service [19, 34, 41], establishing national universal medical insurance [42], and catastrophic disease insurance schemes [25, 27]. Consistent with the literature, the lower level of satisfaction in Northeast China found in this study may be attributed to the vulnerable healthcare system in this region due to the prolonged stagnant economy and tight fiscal budgets of local governments [25, 40].

Despite that high-quality healthcare resources in China are concentrated in urban areas and central cities [9, 27, 34], rural residents in China in recent studies were found to generally report higher levels of satisfaction with the healthcare system [1, 9, 11, 40], owing to the establishment of new rural community health services [1, 11, 43]. The quantile analysis in this study suggests that more attention should be directed to the least satisfied group in rural areas, who are among the disadvantaged groups in vulnerable communities.

The quantile regression analysis in this study also found that there are heterogeneities in satisfaction with the healthcare utilization and access among rural and urban residents in China. Tertiary hospitals in urban areas are generally perceived to have good care quality [6], whereas those in townships have lower grades [26]. Higher hospital beds occupancy in rural areas may be associated with higher likelihood of hospitalization, which especially means more medical expenditure burden and economic costs for rural residents. This finding is consistent with literature reporting that treatment outcome was the strongest predictor of overall satisfaction among rural residents in China [44].

In rural areas, where the healthcare resources were not as abundant, daily visits per physician in public hospitals were positively associated with residents’ satisfaction because they may be satisfied with easier access to healthcare staff and shorter waiting time [1, 11, 43]. However, the daily visit volume had a strong negative association among urban residents consistently across most percentile positions. This heterogeneous response is because tertiary hospitals in cities are highly desired and overcrowded with a high volume of patients [25, 41]. Long waiting time and stressful environments often lead to patient dissatisfaction [10, 45].

The healthcare expenditure ratio was insignificant for most estimations. With the establishment of universal healthcare insurance and economy development in China, the economic burden may not be the major driven factor for healthcare satisfaction. The significant moderate positive association at median may suggest a higher demand and stronger willingness to pay for healthcare in China [9].

The negative association among the rural residents at median and lower percentiles, though statistically insignificant in this study, calls for additional investigation and remedies to further reduce the economic burden of their healthcare expenditure [34, 43, 46].

This study had several limitations, largely due to the availability of data. First, public satisfaction was only measured using a single question in the survey data, without information about a specific aspect of the healthcare system. Second, based on cross-sectional data, this study could only make inferences about associations at various percentiles, although potential causal effects might exist de facto. Third, quality of care was not measured in this study due to data availability. In addition, there may be unidentified confounding factors of satisfaction scores, such as social media influences, which were not included in the survey. Finally, the survey data in this study were collected in 2015, when the early impacts of the 2013 healthcare reform may have been reflected. The findings based on this dataset are still relevant to the current reform and the impacts of the next stage.

## Conclusion

Applying a quantile regression method, this study found that, among the most satisfied residents in China, the healthy groups tend to have lower satisfaction levels with the healthcare system. Meanwhile, the “pro-rich” inequality was especially significant among those with  lower levels of satisfaction (at or below the median). Providing detailed information about disparities and socioeconomic inequality in residents’ satisfaction with the healthcare system in China, the findings of this study may help to enhance the precision or policy targeting of the healthcare reform. First, to meet the healthcare needs of the healthy residents, the healthcare system needs to further incorporate preventive care and service, which are highly cost-effective in the long run too. Second, the disparities associated with higher socioeconomic status or the “pro-rich” inequality in the healthcare system in China should receive serious attention and correction actions during the Common Prosperity campaign. Third, more attention and supports need to be channeled to the healthcare system in Northeast China, which is vulnerable largely because of the prolonged stagnant economy, shrinking population and healthcare professionals migrating to other developed cities in the country. Finally, in addition to healthcare access and utilization, outcome-based high-quality care is preferred by rural residents and quality enhancement should be the center of healthcare equality improvement in rural China.

## Data Availability

Data available in a publicly accessible repository: (1) The Chinese General Social Survey (CGSS), a national representative continuous survey project available in China since 2003, is publicly downloadable at http://www.cnsda.org/index.php. The public health satisfaction score data of CGSS2015 are up to date. (2) Data on healthcare resources and expenditure at the provincial level were obtained from the China Public Health Statistical Yearbook 2015, accessible through subscription-based databases.
